# Supplementary material for: Sensible heat has significantly affected the global hydrological cycle over the historical period
Source: Nat Commun. 2018 May 15;9:1922. doi: 10.1038/s41467-018-04307-4 (PMC5954152; doi:10.1038/s41467-018-04307-4)
Supplement: Supplementary file 1 — Supplementary Information [file 41467_2018_4307_MOESM1_ESM.pdf]

Supplementary Information for manuscript "Sensible heat has significantly affected the global hydrological cycle over the historical period"

By Myhre et al.

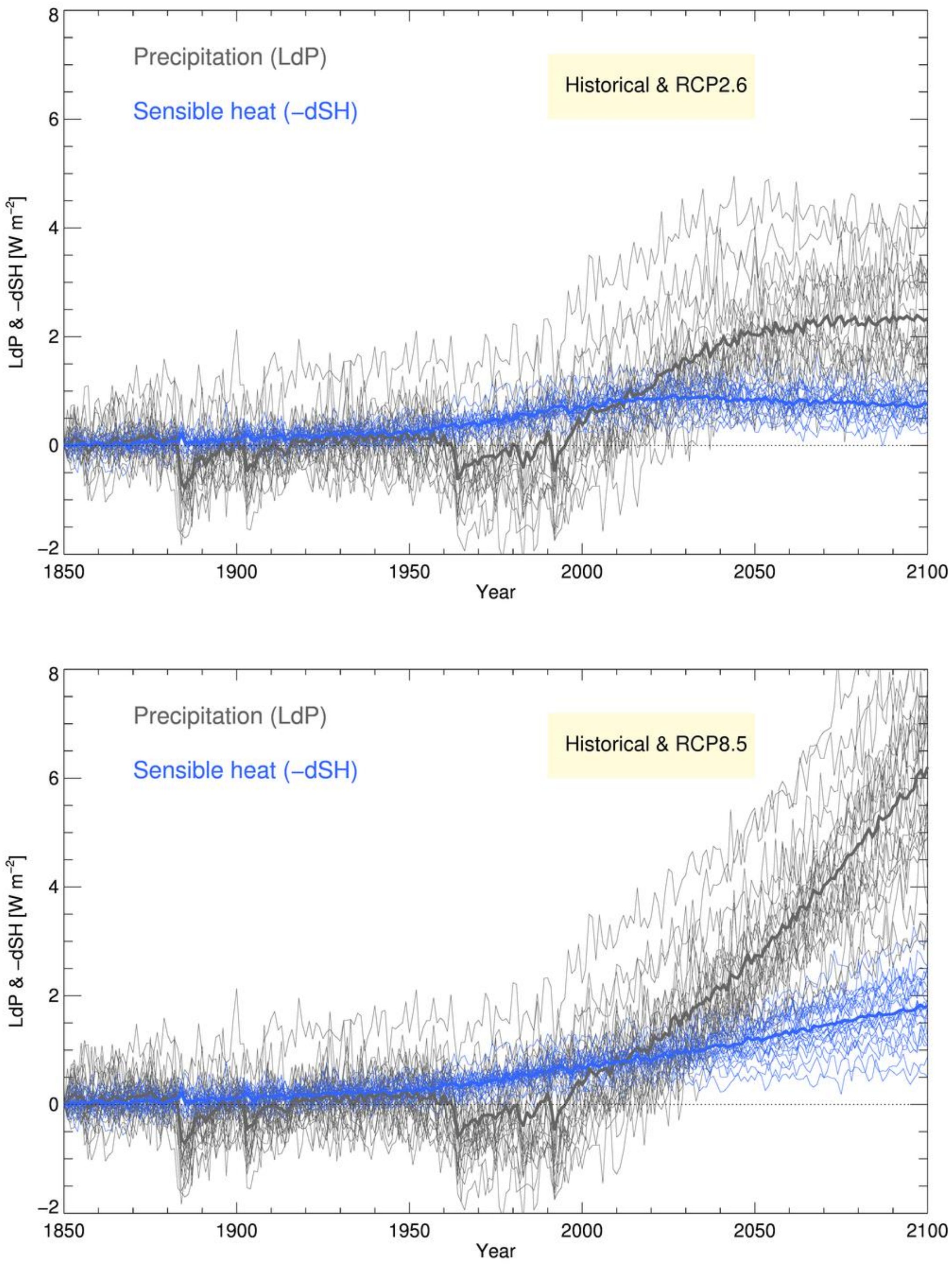

Supplementary Figure 1: Global historical and future precipitation and sensible heat changes from CMIP5 models based on RCP2.6 (upper panel) and RCP8.5 (lower panel) as shown in Figure 2 with each model shown as a separate thin line. The multi-model means are shown with thick lines.

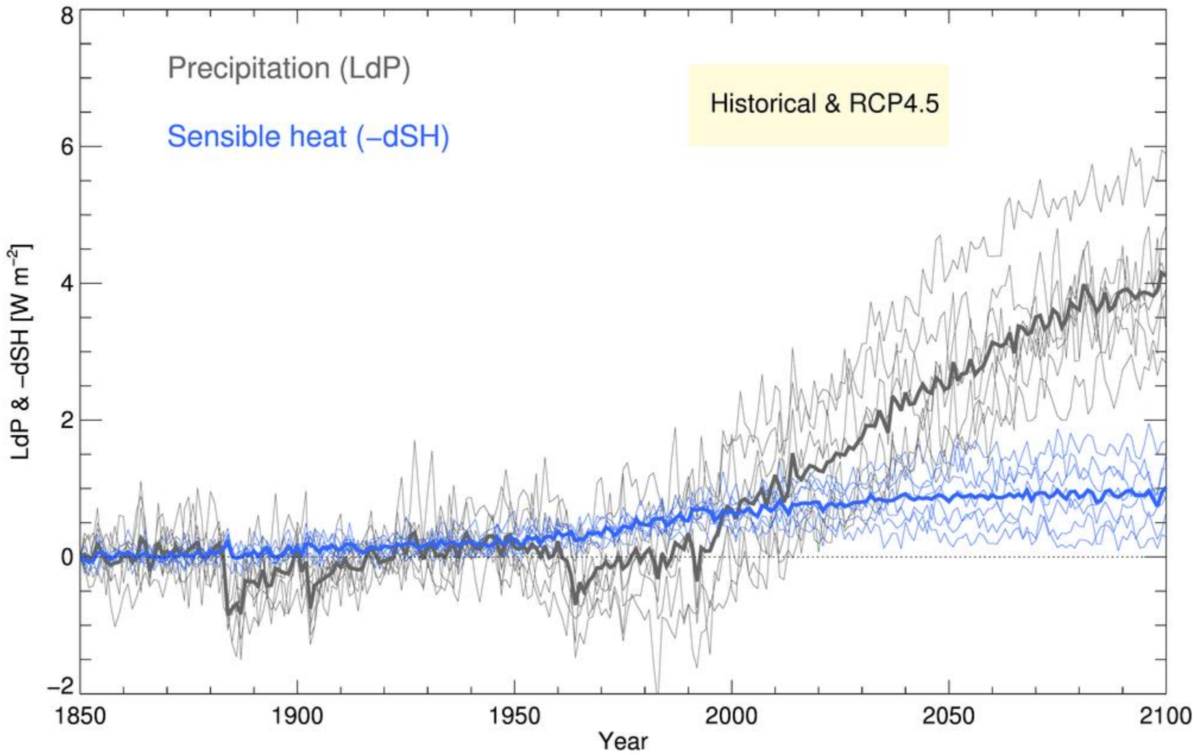

Supplementary Figure 2: Global historical and future precipitation and sensible heat changes from a subset of CMIP5 models. Future simulations are based on RCP4.5. Sensible heat is given as a reduction in the surface flux and thus is a contribution to precipitation according to Equation 1.

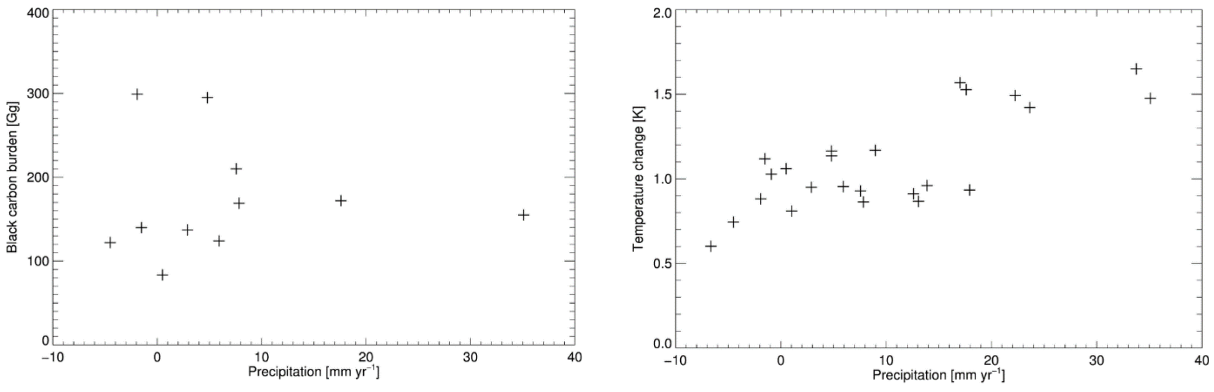

Supplementary Figure 3: Precipitation changes from CMIP5 models versus black carbon burden (left panel) and surface temperature changes (right panel). Precipitation changes are taken between 2010-2016 versus 1861-1867 for the 24 CMIP5 models given in Table S6 in the panel right) whereas in panel left) the 11 models overlapping between models given in Table S6 and black carbon burden in 2010 given for CMIP5 models in Allen and Landuyt [1]. CMIP5 surface temperature change is calculated for the same period as the precipitation change. The correlation of data given in the left panel) is -0.03 and in the right panel) is 0.76.

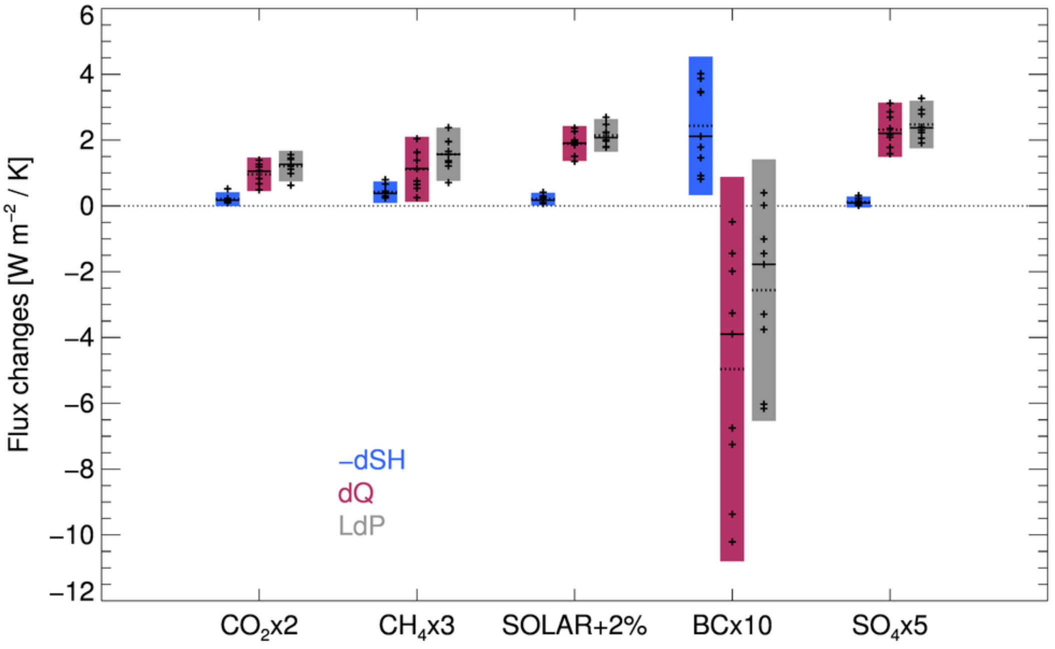

Supplementary Figure 4: Changes in sensible heat (plotted as the negative of the change, -dSH), radiative cooling (dQ), and precipitation (LdP) for five drivers of climate change from PDRMIP normalized by global mean temperature change. See further description in Figure 4 in the main manuscript for details.

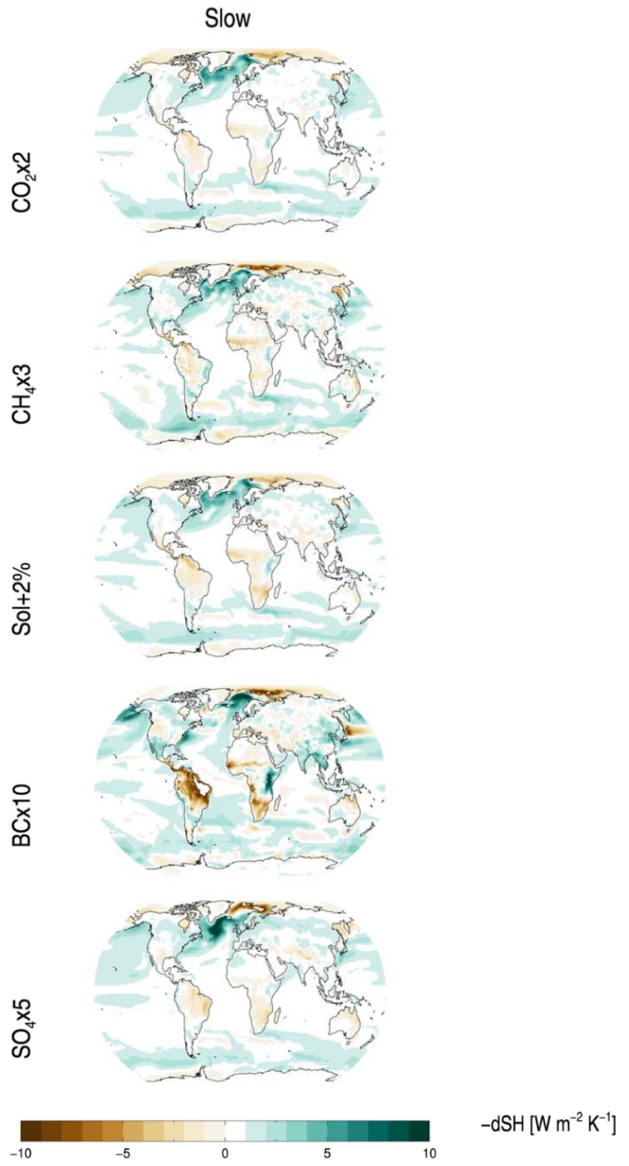

Supplementary Figure 5: Changes in slow surface sensible heat normalized by global mean surface temperature change for five drivers of climate change (in order from top to bottom: CO_2x2, CH_4x3, Solar+2%, BCx10, SO_4x5, see Methods for further description). Note that the figure shows the reduction of sensible heat from the surface to the atmosphere and the contribution of sensible heat to increase in precipitation and thus –dSH according to Equation 1. Results are shown for the multi-model mean of the PDRMIP models.

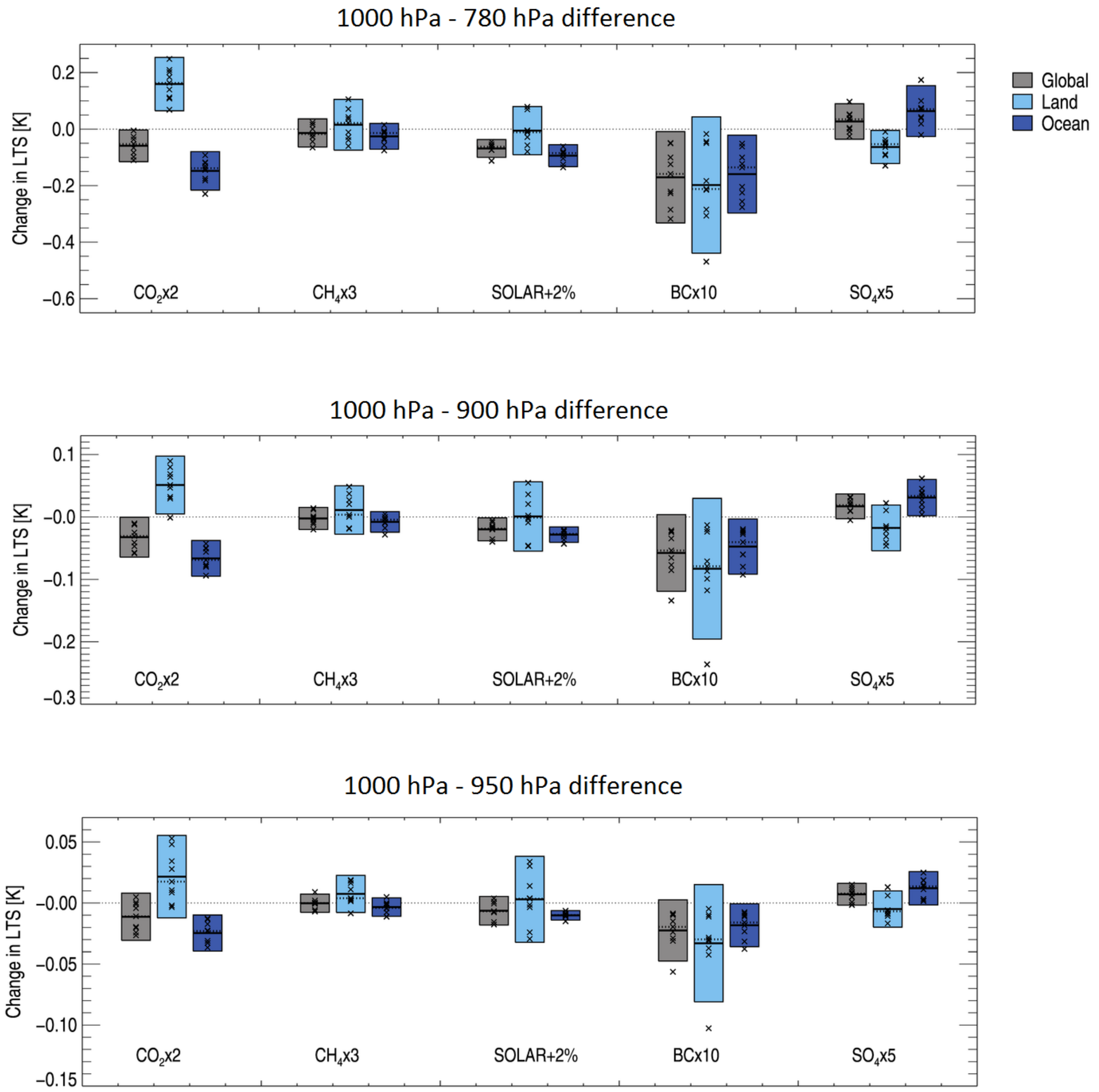

Supplementary Figure 6: Lower tropospheric stability (LTS) for three different choices of the pressure of the upper level for the five PDRMIP climate drivers. The upper panel shows the change in LTS between the 1000 hPa and 780 hPa layers identical to shown in Figure 6. The middle and lower panel show the change in LTS between 1000 hPa and 900 hPa and 1000 hPa and 950 hPa, respectively. Surface pressure can at some locations be lower than 1000 hPa, especially at high altitudes over land. The 1000 hPa level represent the lowest model layers whereas the upper level given in the three panels is the pressure level for a given surface pressure of 1000 hPa. The individual PDRMIP models are shown with crosses and mean and median values shown with horizontal dashed and solid lines, respectively.

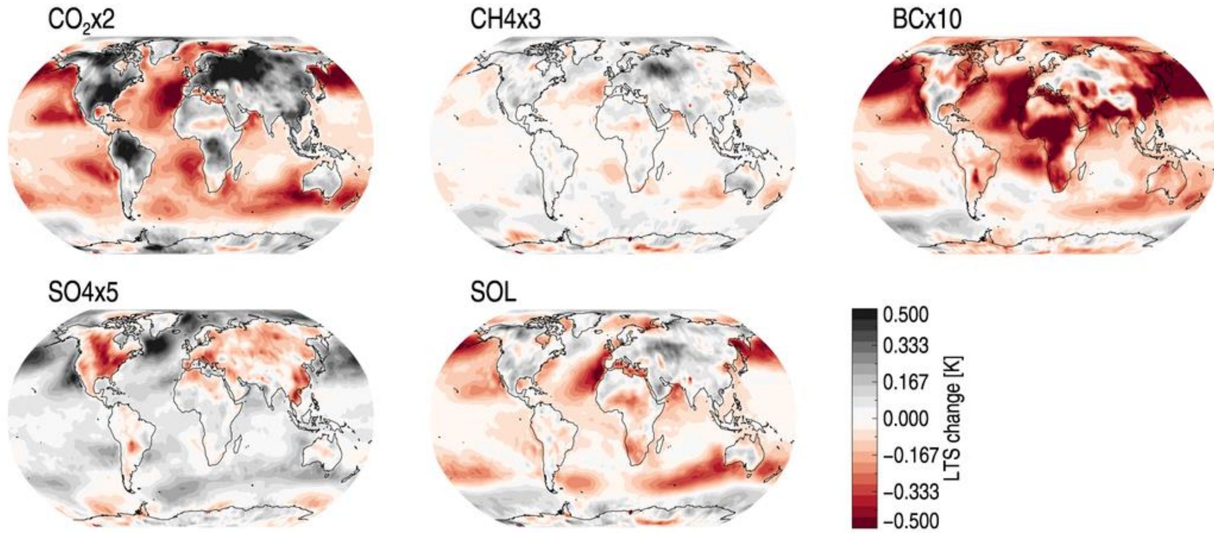

Supplementary Figure 7: Multi-model mean LTS changes for the five PDRMIP drivers. LTS is here defined as the temperature in the vertical layer corresponding to 1000hPa minus the temperature in the 780hPa-layer, based on years 6-15 in fixed-SST simulations and similar to Figure 6 in the main manuscript.

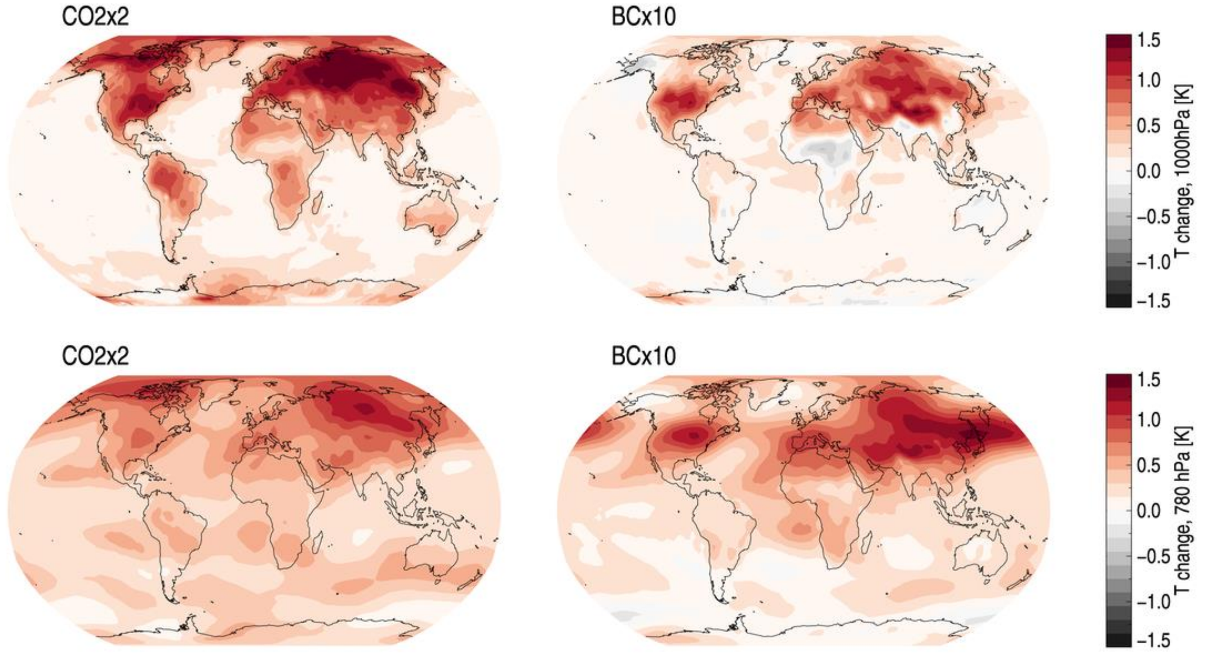

Supplementary Figure 8: Multi-model mean PDRMIP temperature change at 1000 hPa and 780 hPa for CO2x2 and BCx10, based on years 6-15 in fixed-SST simulations.

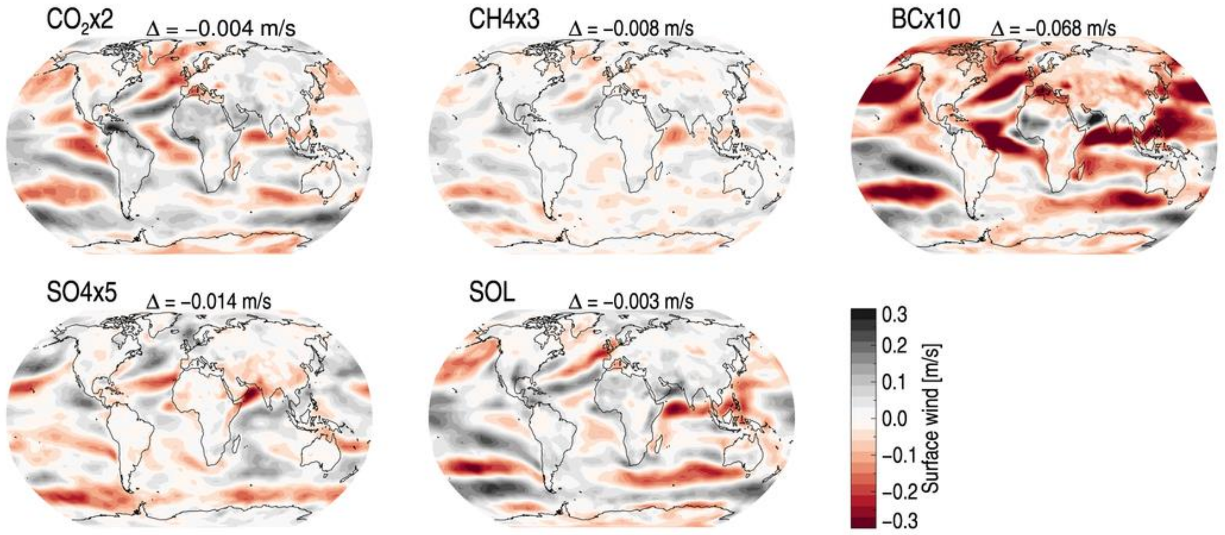

Supplementary Figure 9: Multi-model mean near surface wind changes for the five PDRMIP drivers from the fixed-SST simulations.

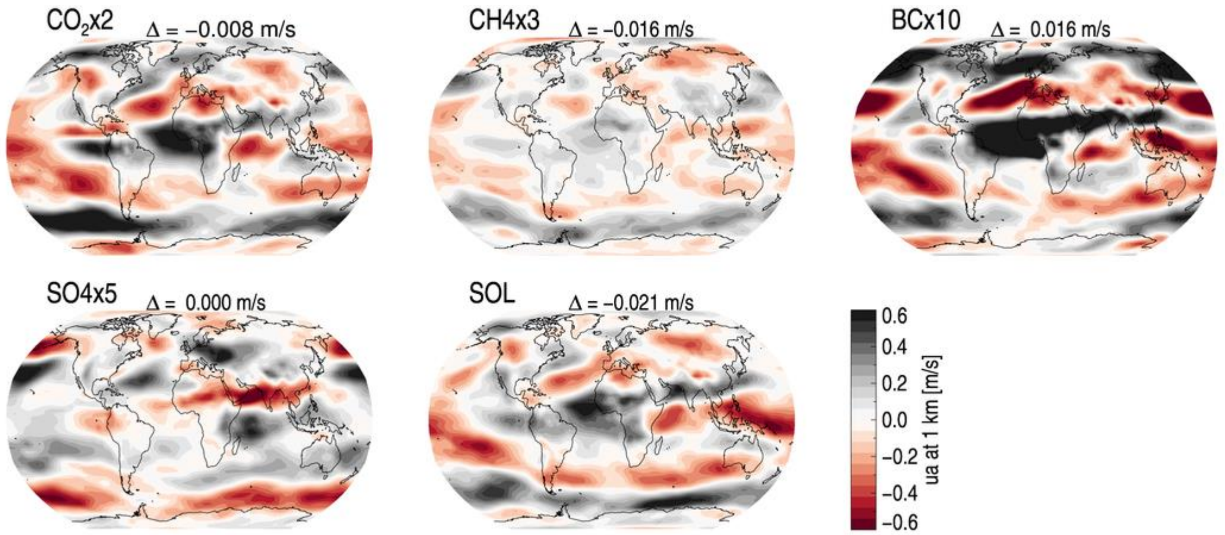

Supplementary Figure 10: Multi-model mean changes in zonal wind at 1 km for the five PDRMIP drivers from the fixed-SST simulations.

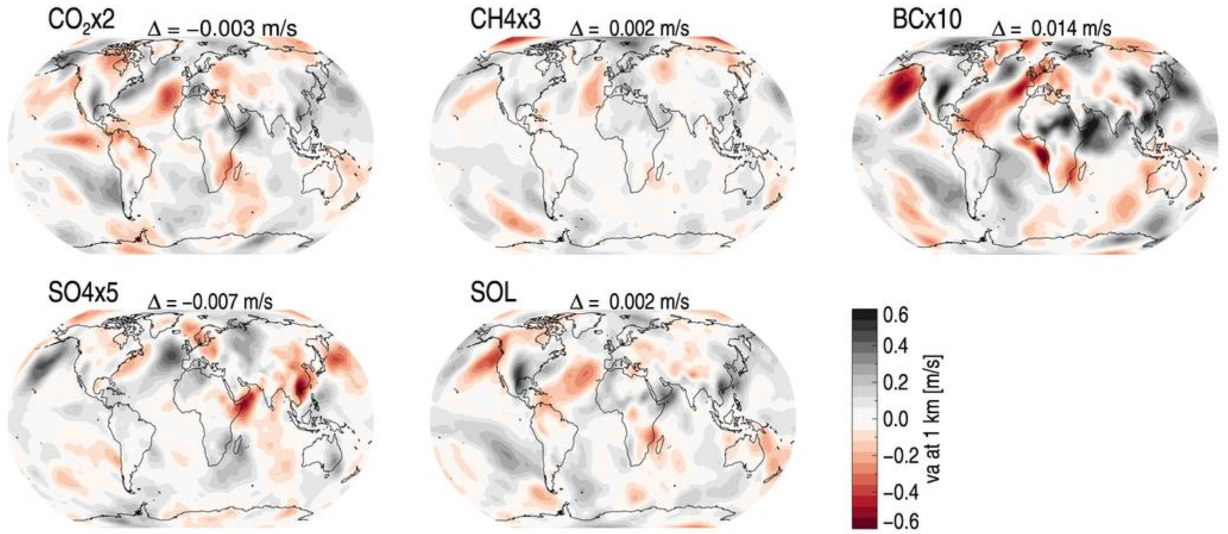

Supplementary Figure 11: Multi-model mean changes in meridional wind at 1 km for the five PDRMIP drivers from the fixed-SST simulations.

Supplementary Table 1: Mean of nine PDRMIP models for top of the atmosphere forcing and fast sensible heat changes for the five PDRMIP drivers. Fast sensible heat changes are given as –dSH according to Equation 1.

	dF_TOA_fast (Wm^{-2})	-dSH fast (Wm^{-2})
CO_2x2	3.65	-0.01
CH_4x3	1.20	0.14
Solar	4.18	-0.02
BCx10	0.97	1.14
SO_4x5	-3.53	0.24

Supplementary Table 2: Calculations of sensible heat changes (slow changes, based on historical temperature changes in CMIP5 models) and fast from IPCC AR5 forcing normalized to PDRMIP results. Sensible heat changes given as –dSH according to Equation 1.

Slow SH	Change (K)	-dSH (Wm^{-2})	Comment
dT	1.09 K	0.25	0.23 W m^{-2} K^{-1} for -dSHslow / dT as mean of PDRMIP drivers (CO_2 – 0.23, CH_4 – 0.19, Sol – 0.23, Sul – 0.26). BC is weaker (0.04 W m^{-2} K^{-1}) , but warming from BC in these simulations is small.
**Fast SH**	**Historical forcing[2] (Wm^{-2})**	**-dSH (Wm^{-2})**	**Comment**
CO_2	1.89	0.00	A 3% higher forcing than in AR5 is used to represent the 2010-2016 period.
CH_4	0.48	0.06	
N_2O	0.17	0.02	Assume dSHfast similar to CH_4 so scaling performed relative to the CH4x3 case.
Halocarbons	0.35	0.04	Same as for N_2O, we have scaled this to the CH4x3 case.
Ozone	0.35	0.01	A weak change in dSHfast is assumed based on MacIntosh et al.[3]
Surface albedo	-0.15	0.00	We lack information on this case and therefore assumed a zero value. The forcing is, in any case, small compared to other components.
BC		0.11	10% of BCx10 and unlike the other drivers scaling to IPCC AR5 numbers not needed. Using BC from PDRMIP is more consistent with CMIP5 models than scaling to IPCC AR5 numbers. The historical forcing according to the PDRMIP models would be 0.097 W m^{-2}.
Aerosols	-1.5	0.10	Total scattering aerosols derived as the difference between total aerosol forcing minus BC from IPCC AR5.
Total fast -dSH		0.34	
**Total -dSH**		**0.59***	

*PDRMIP models have lower SH changes than CMIP5 mean by 12% and results in Figure 8 scaled to take this into account.

Supplementary Table 3: Mean of nine PDRMIP models for atmospheric radiative heating (-dQfast), atmospheric radiative cooling normalized by global mean temperature (dQslow) for the PDRMIP drivers.

	RF_Atm_fast ($W\ m^{-2}$)	dQ slow/dT ($Wm^{-2}\ K^{-1}$)
CO_2x2	2.19	1.93
CH_4x3	0.58	2.19
Solar	0.61	2.20
BCx10	3.25	2.01
SO_4x5	0.29	2.06

Supplementary Table 4: Calculations of atmospheric radiative cooling changes (slow based on historical temperature changes and fast from IPCC AR5 forcing normalized to PDRMIP results). The scaling for the climate drivers not included in the PDRMIP core simulations, the same assumption for the scaling is applied as in Supplementary Table 2.

Slow dQ	Change (K)	dQ ($W\ m^{-2}$)	Comment
**dT**	1.09	**2.21**	A value of 2.03 $W\ m^{-2}\ K^{-1}$ is adopted from Supplementary Table 3
Fast dQ	Historical forcing[2] ($W\ m^{-2}$)	dQ ($W\ m^{-2}$)	
CO_2	1.89	-1.13	
CH_4	0.48	-0.23	
N_2O	0.17	-0.08	
Halocarbon	0.35	-0.17	
Ozone	0.35	-0.00	
Surface albedo	-0.15	-0.00	
BC		-0.32	
Aerosols	-1.5	-0.12	
**Total fast dQ**		**-2.06**	

Supplementary Table 5: Relative standard deviation (RSD; defined as the standard deviation among the models divided by the multi-model mean) from PDRMIP models for sensible heat (total), fast atmospheric heating, radiative cooling caused by surface and tropospheric temperature changes (slow). All are in units of Wm^{-2}.

	Fast atmospheric heating	Sensible heat	Slow radiative cooling divided by temperature
CO_2x2	14.6	-70.4	11.0
CH_4x3	53.6	-27.8	16.8
Solar	32.2	-63.4	17.1
BCx10	39.5	-43.8	52.0
SO_4x5	123.2	120.9	18.2

Supplementary Table 6: CMIP5 models included in this work for historical and future analysis. Crosses indicate model data was available. Model names given in bold are also **PDRMIP** models.

Model	Historical	RCP2.6	RCP8.5
ACCESS1-0	X		X
ACCESS1-3	X		X
BCC-CSM1.1-M	X	X	X
BNU-ESM	X	X	X
**CanESM**	X	X	X
**CCSM4**	X	X	X
**CESM1-CAM5**	X	X	X
CMCC-CMS	X		X
CNRM-CM5	X	X	X
CSIRO-Mk3.6	X	X	X
FGOALs-g2	X	X	X
FIO-ESM	X	X	X
GFDL-CM3	X	X	X
GFDL-ESM2M	X	X	X
**GISS-ER-R**	X	X	X
**HadGEM2-ES**	X	X	X
INM-CM4	X		X
**IPSL-CM5A-LR**	X	X	X
MIROC5	X	X	X
**MIROC-ESM**	X	X	X
**MPI-ESM-LR**	X	X	X
MRI-CGCM3	X	X	X
MRI-ESM1	X		X
**NorESM1-M**	X	X	X

1 Allen, R. J. and Landuyt, W., The vertical distribution of black carbon in CMIP5 models: Comparison to observations and the importance of convective transport. *J. Geophys. Res. - Atmos.* **119**, 4808-4835 (2014).

2 Myhre, G. et al., *Anthropogenic and Natural Radiative Forcing*, in *Climate Change 2013: The Physical Science Basis. Contribution of Working Group I to the Fifth Assessment Report of the Intergovernmental Panel on Climate Change*, edited by T. F. Stocker et al. (Cambridge University Press, Cambridge, United Kingdom and New York, NY, USA, 2013), pp. 659-740.

3 MacIntosh, C. R. et al., Contrasting fast precipitation responses to tropospheric and stratospheric ozone forcing. *Geophys. Res. Lett.* **43**, 1263-1271 (2016).
